# Supplementary material for: A Humanized Yeast Phenomic Model of Deoxycytidine Kinase to Predict Genetic Buffering of Nucleoside Analog Cytotoxicity
Source: Genes (Basel). 2019 Sep 30;10(10):770. doi: 10.3390/genes10100770 (PMC6826991; doi:10.3390/genes10100770)
Supplement: Supplementary file 1 [file genes-10-00770-s001.zip › Additional_Files/Additional_File7_GOTermHeatmaps.zip!/Additional_File7_GOTermHeatmaps/protein-DNA_complex.pdf]

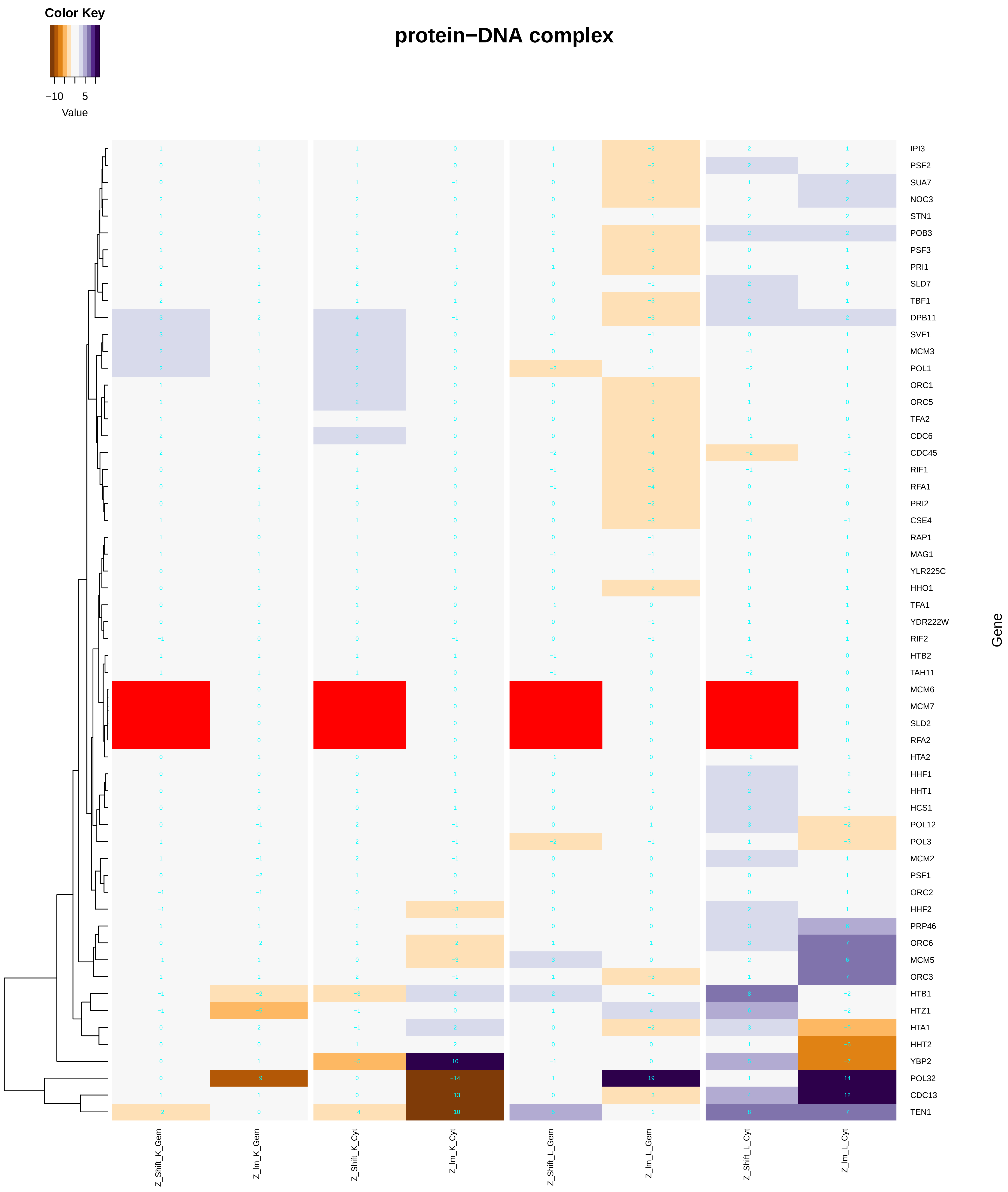

Color Key
−10
5
Value
protein−DNA complex
IPI3
PSF2
SUA7
NOC3
STN1
POB3
PSF3
PRI1
SLD7
TBF1
DPB11
SVF1
MCM3
POL1
ORC1
ORC5
TFA2
CDC6
CDC45
RIF1
RFA1
PRI2
CSE4
RAP1
MAG1
YLR225C
HHO1
TFA1
YDR222W
RIF2
HTB2
TAH11
MCM6
MCM7
SLD2
RFA2
HTA2
HHF1
HHT1
HCS1
POL12
POL3
MCM2
PSF1
ORC2
HHF2
PRP46
ORC6
MCM5
ORC3
HTB1
HTZ1
HTA1
HHT2
YBP2
POL32
CDC13
TEN1
Gene
Z_Shift_K_Gem
Z_Im_K_Gem
Z_Shift_K_Cyt
Z_Im_K_Cyt
Z_Shift_L_Gem
Z_Im_L_Gem
Z_Shift_L_Cyt
Z_Im_L_Cyt

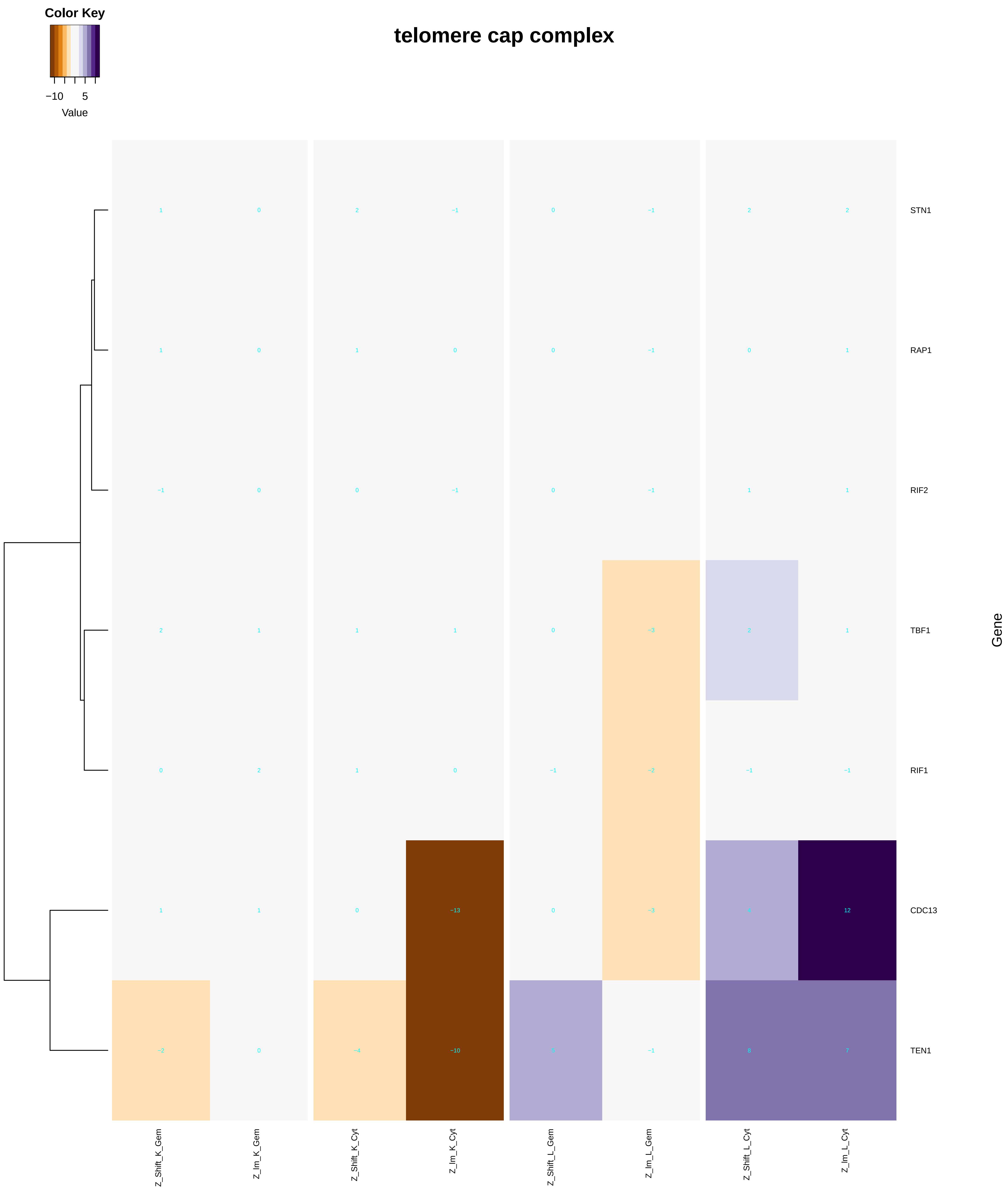
Color Key
−10
5
Value
telomere cap complex
STN1
RAP1
RIF2
TBF1
RIF1
CDC13
TEN1
Gene
Z_Shift_K_Gem
Z_Im_K_Gem
Z_Shift_K_Cyt
Z_Im_K_Cyt
Z_Shift_L_Gem
Z_Im_L_Gem
Z_Shift_L_Cyt
Z_Im_L_Cyt

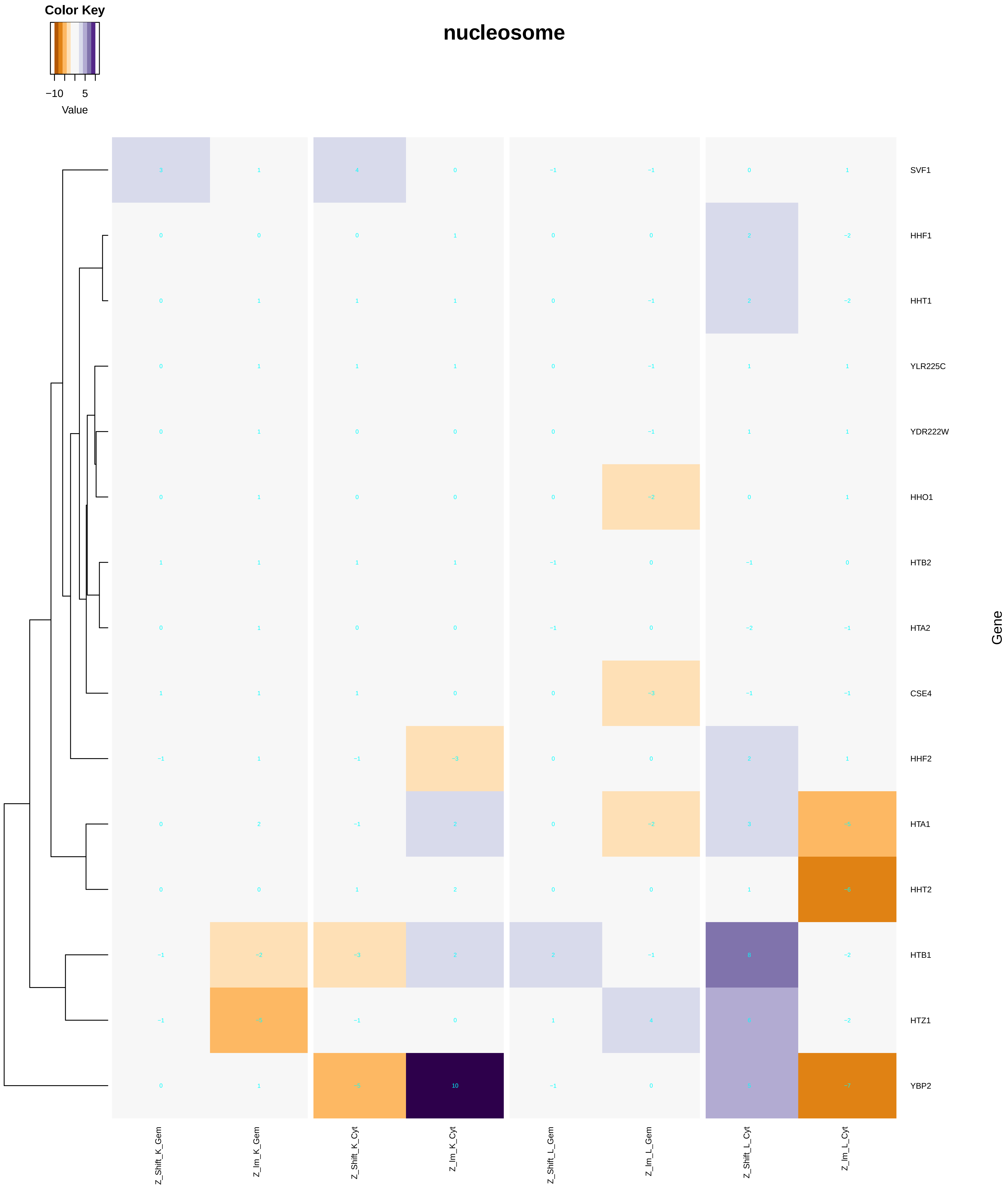
Color Key
−10
5
Value
nucleosome
SVF1
HHF1
HHT1
YLR225C
YDR222W
HHO1
HTB2
HTA2
CSE4
HHF2
HTA1
HHT2
HTB1
HTZ1
YBP2
Gene
Z_Shift_K_Gem
Z_Im_K_Gem
Z_Shift_K_Cyt
Z_Im_K_Cyt
Z_Shift_L_Gem
Z_Im_L_Gem
Z_Shift_L_Cyt
Z_Im_L_Cyt

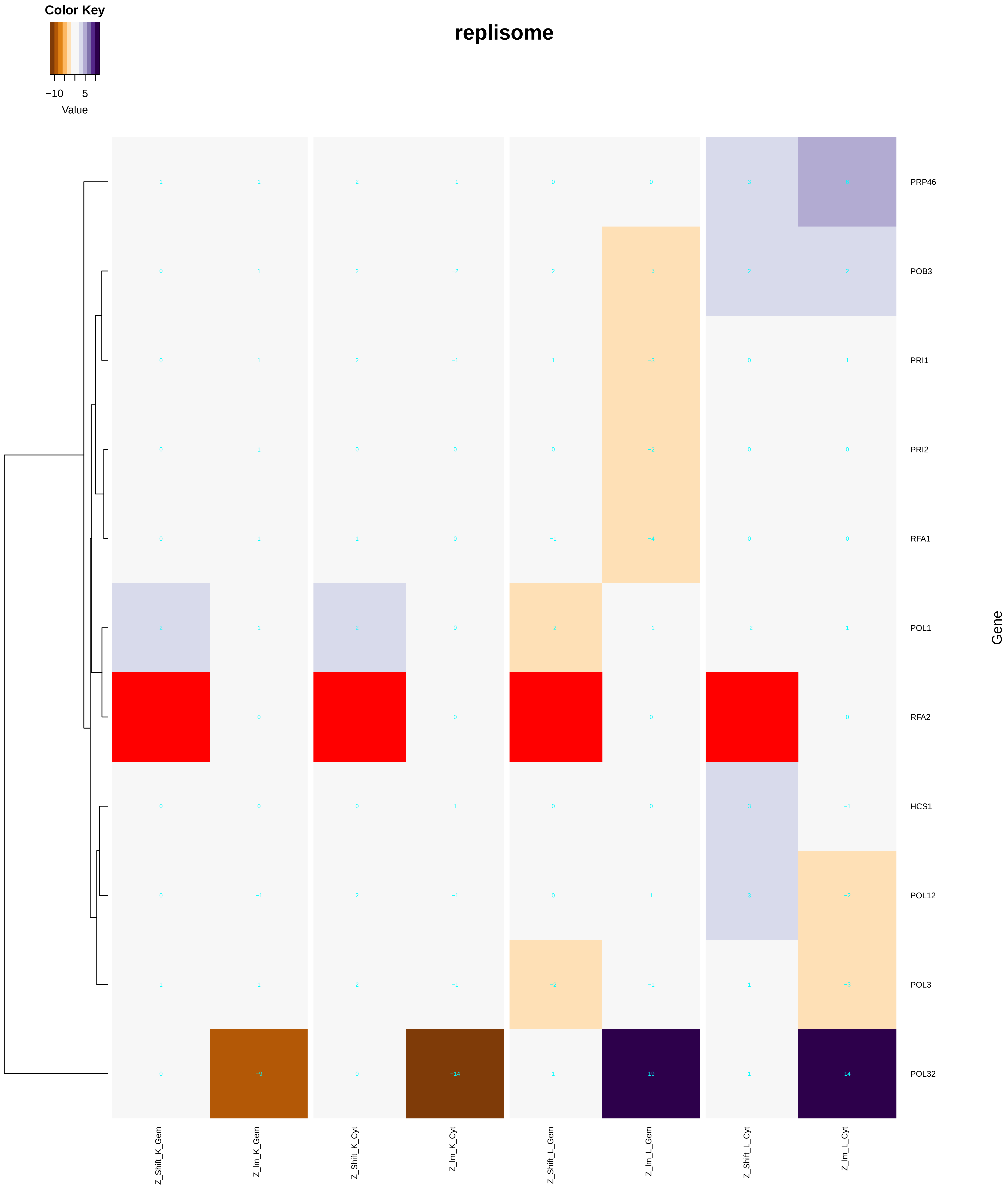
Color Key
−10
5
Value
replisome
PRP46
POB3
PRI1
PRI2
RFA1
POL1
RFA2
HCS1
POL12
POL3
POL32
Gene
Z_Shift_K_Gem
Z_Im_K_Gem
Z_Shift_K_Cyt
Z_Im_K_Cyt
Z_Shift_L_Gem
Z_Im_L_Gem
Z_Shift_L_Cyt
Z_Im_L_Cyt

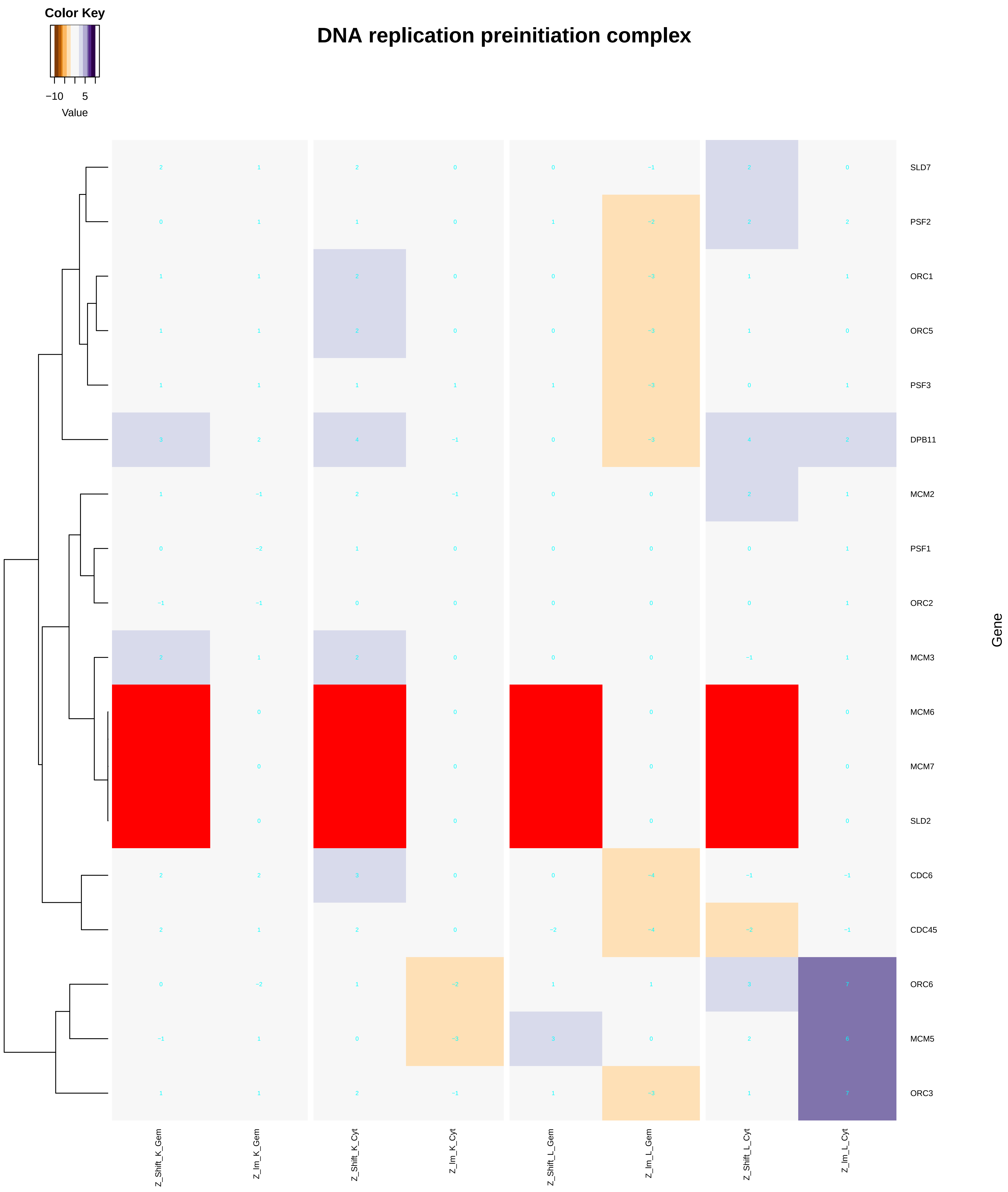

Color Key
−10
5
Value
DNA replication preinitiation complex
SLD7
PSF2
ORC1
ORC5
PSF3
DPB11
MCM2
PSF1
ORC2
MCM3
MCM6
MCM7
SLD2
CDC6
CDC45
ORC6
MCM5
ORC3
Gene
Z_Shift_K_Gem
Z_Im_K_Gem
Z_Shift_K_Cyt
Z_Im_K_Cyt
Z_Shift_L_Gem
Z_Im_L_Gem
Z_Shift_L_Cyt
Z_Im_L_Cyt

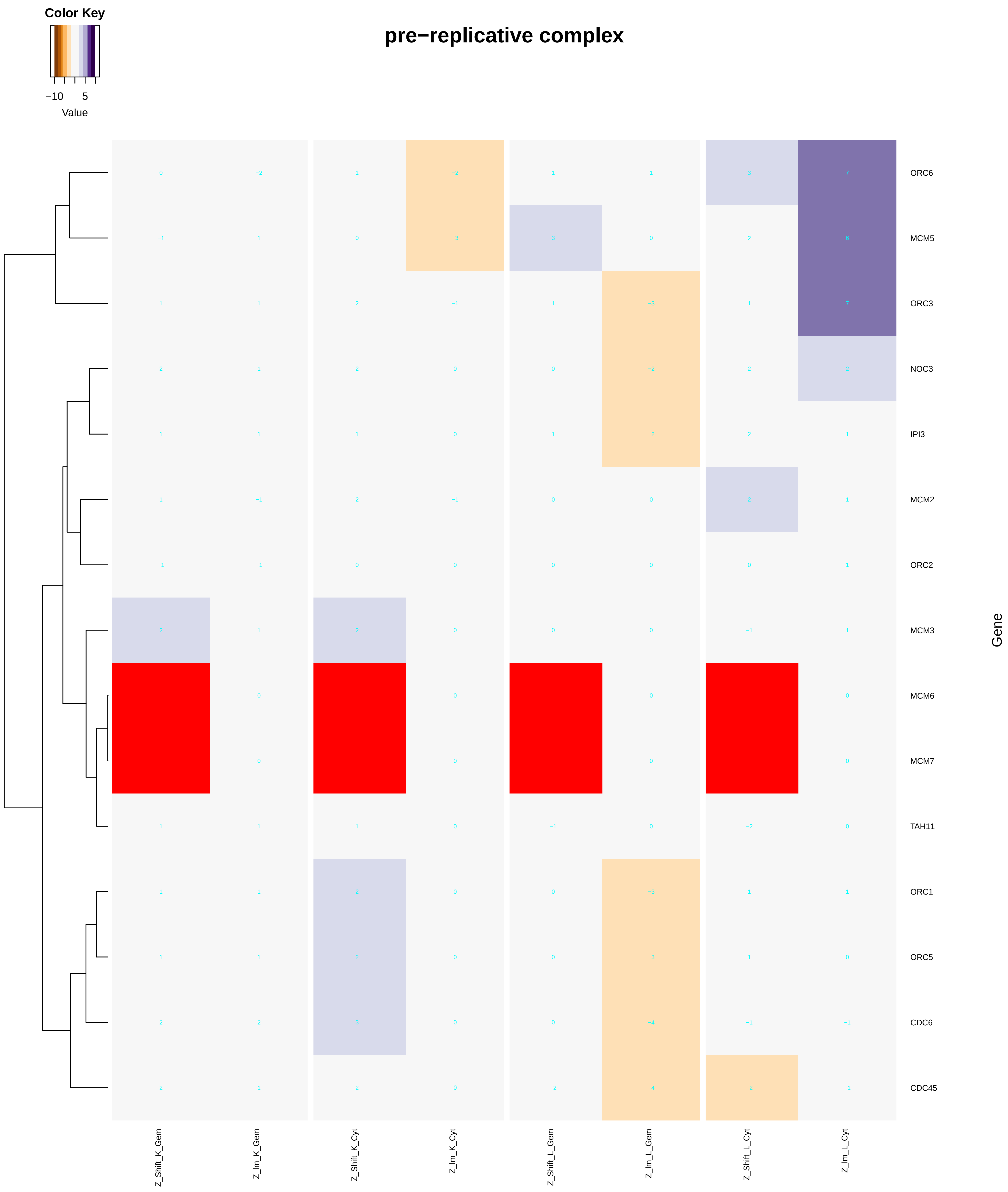
Color Key
−10
5
Value
pre−replicative complex
ORC6
MCM5
ORC3
NOC3
IPI3
MCM2
ORC2
MCM3
MCM6
MCM7
TAH11
ORC1
ORC5
CDC6
CDC45
Gene
Z_Shift_K_Gem
Z_Im_K_Gem
Z_Shift_K_Cyt
Z_Im_K_Cyt
Z_Shift_L_Gem
Z_Im_L_Gem
Z_Shift_L_Cyt
Z_Im_L_Cyt

transcriptional preinitiation complex

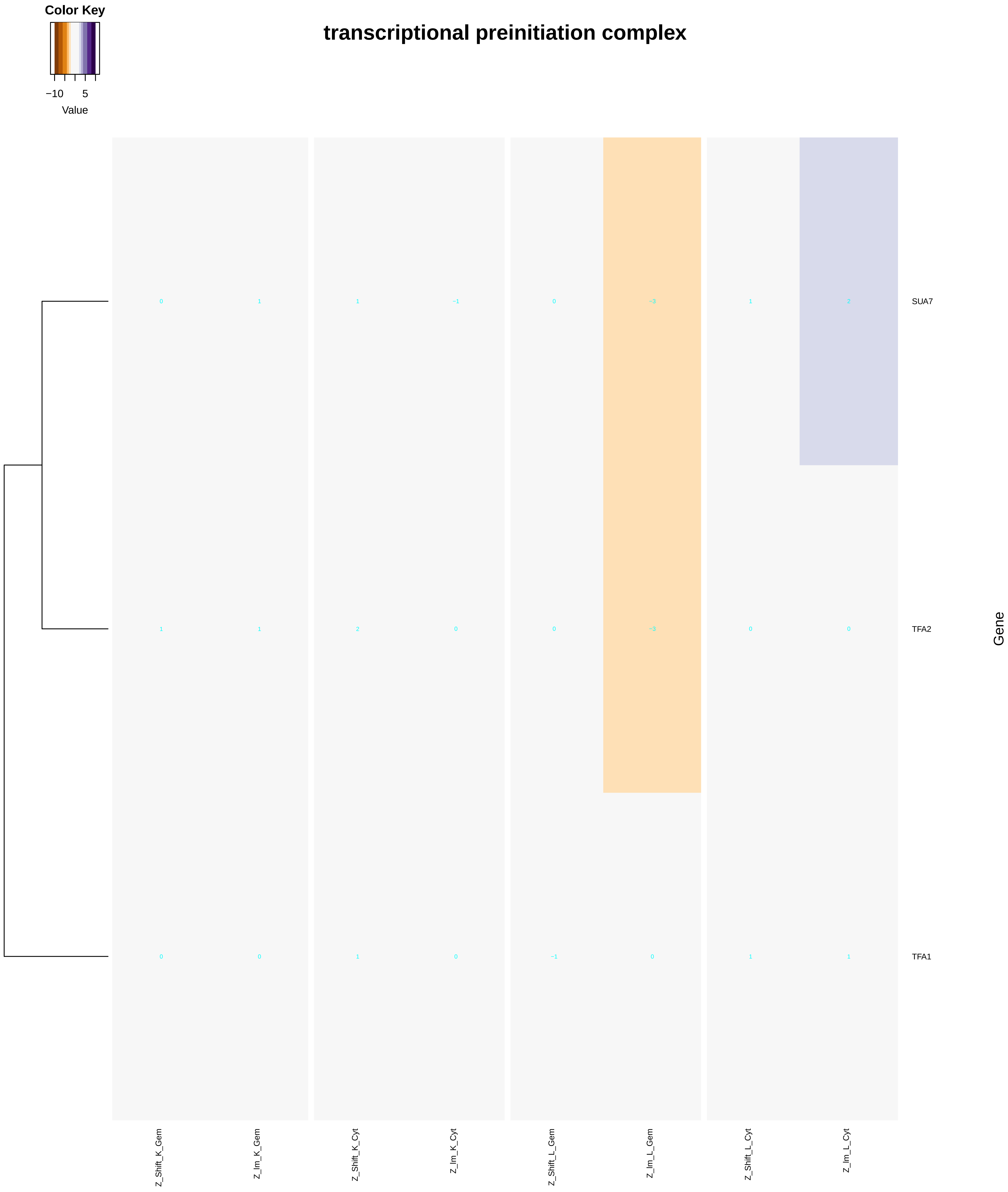

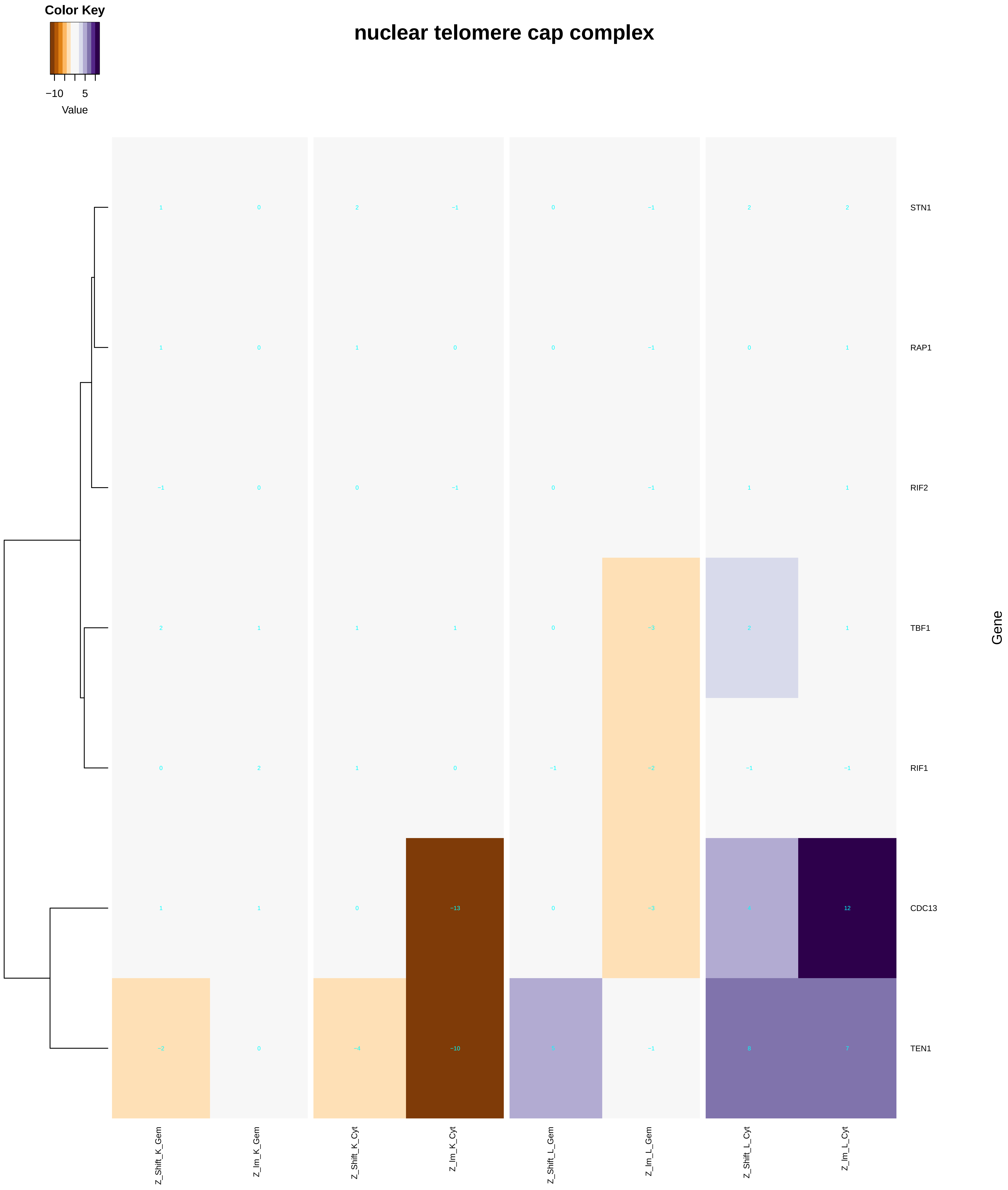

Color Key
−10
5
Value
nuclear telomere cap complex
STN1
RAP1
RIF2
TBF1
RIF1
CDC13
TEN1
Gene
Z_Shift_K_Gem
Z_Im_K_Gem
Z_Shift_K_Cyt
Z_Im_K_Cyt
Z_Shift_L_Gem
Z_Im_L_Gem
Z_Shift_L_Cyt
Z_Im_L_Cyt

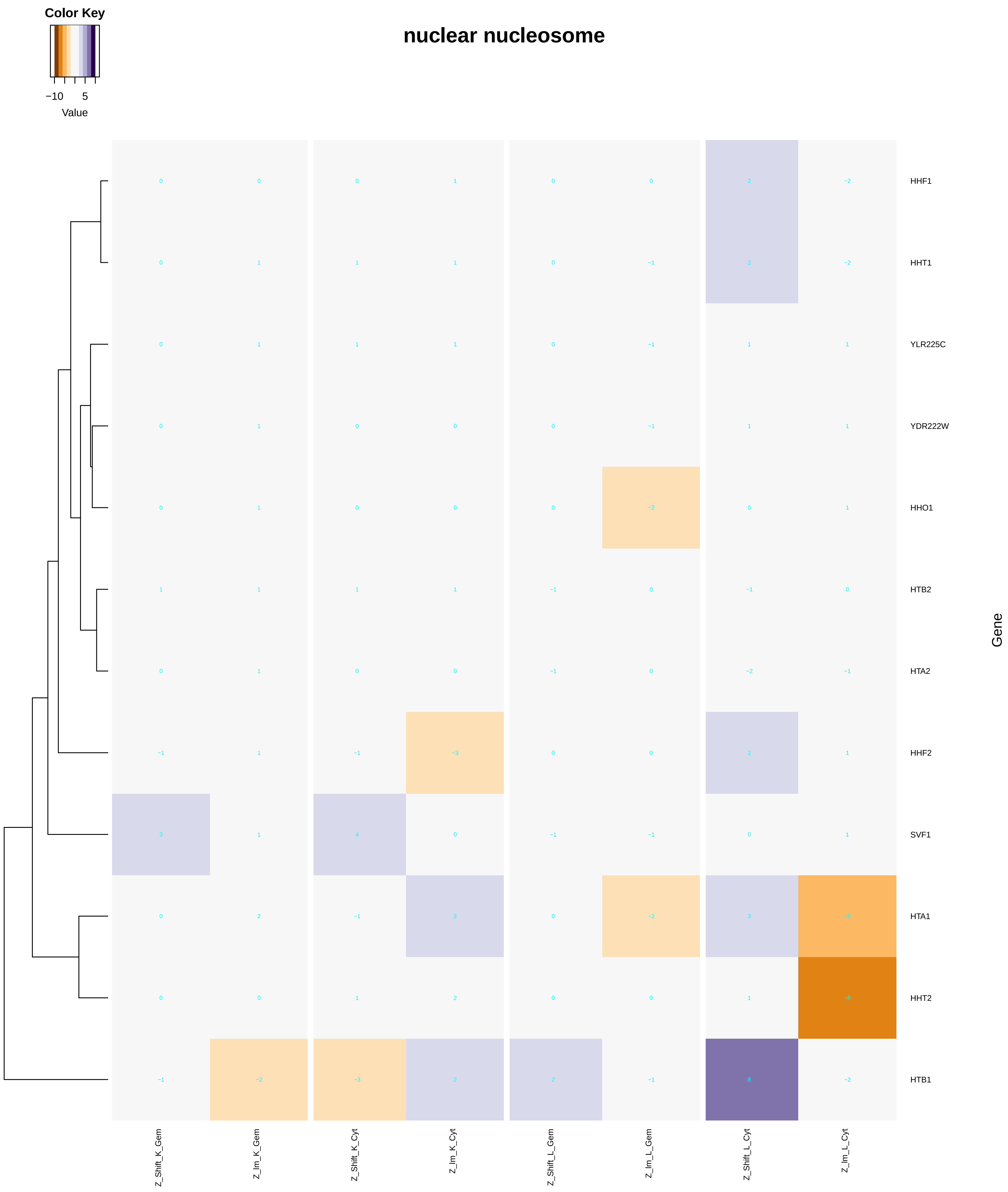
Color Key
−10
5
Value
nuclear nucleosome
HHF1
HHT1
YLR225C
YDR222W
HHO1
HTB2
HTA2
HHF2
SVF1
HTA1
HHT2
HTB1
Gene
Z_Shift_K_Gem
Z_Im_K_Gem
Z_Shift_K_Cyt
Z_Im_K_Cyt
Z_Shift_L_Gem
Z_Im_L_Gem
Z_Shift_L_Cyt
Z_Im_L_Cyt

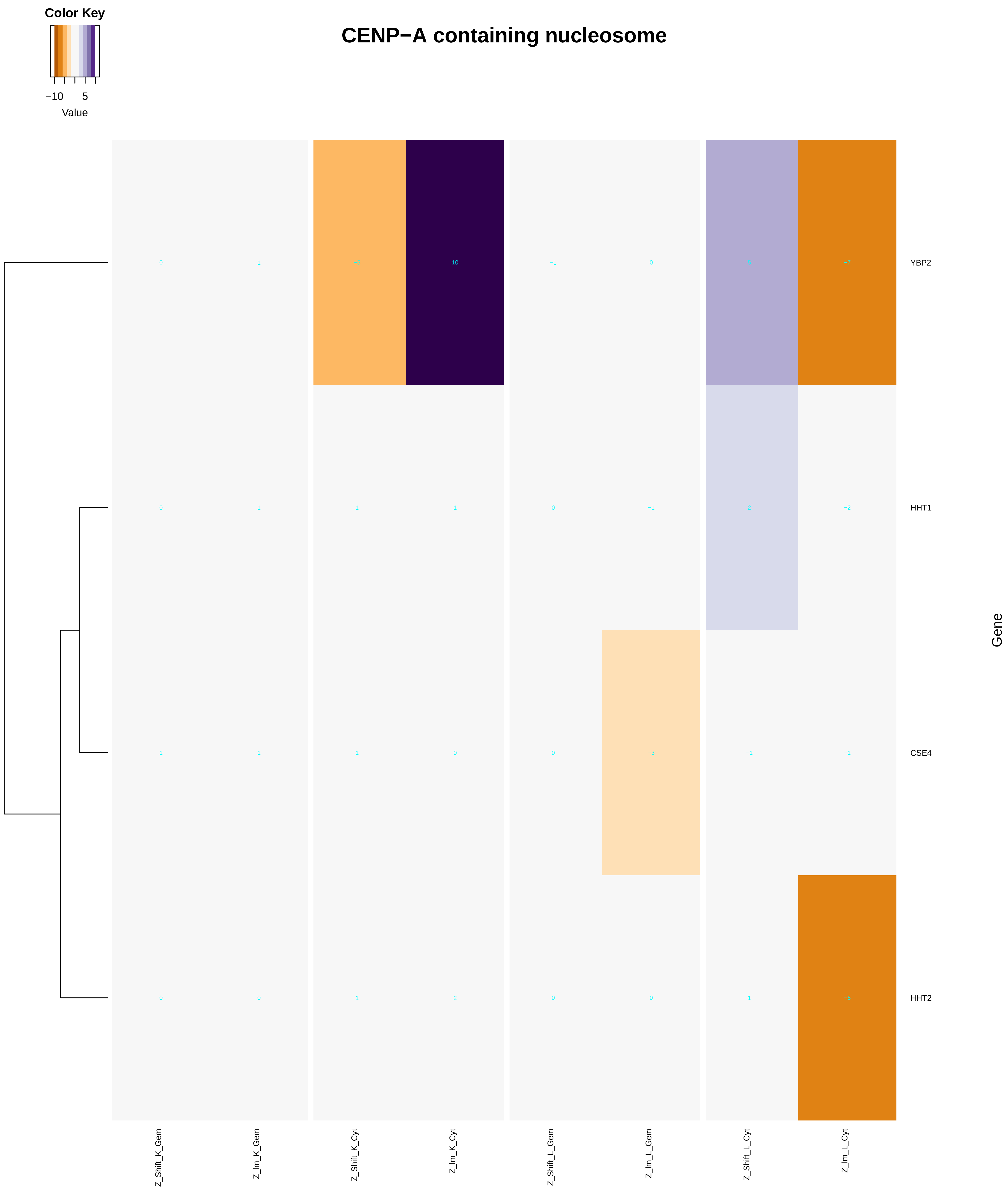

Color Key
−10
5
Value
CENP−A containing nucleosome
YBP2
HHT1
CSE4
HHT2
Gene
Z_Shift_K_Gem
Z_Im_K_Gem
Z_Shift_K_Cyt
Z_Im_K_Cyt
Z_Shift_L_Gem
Z_Im_L_Gem
Z_Shift_L_Cyt
Z_Im_L_Cyt

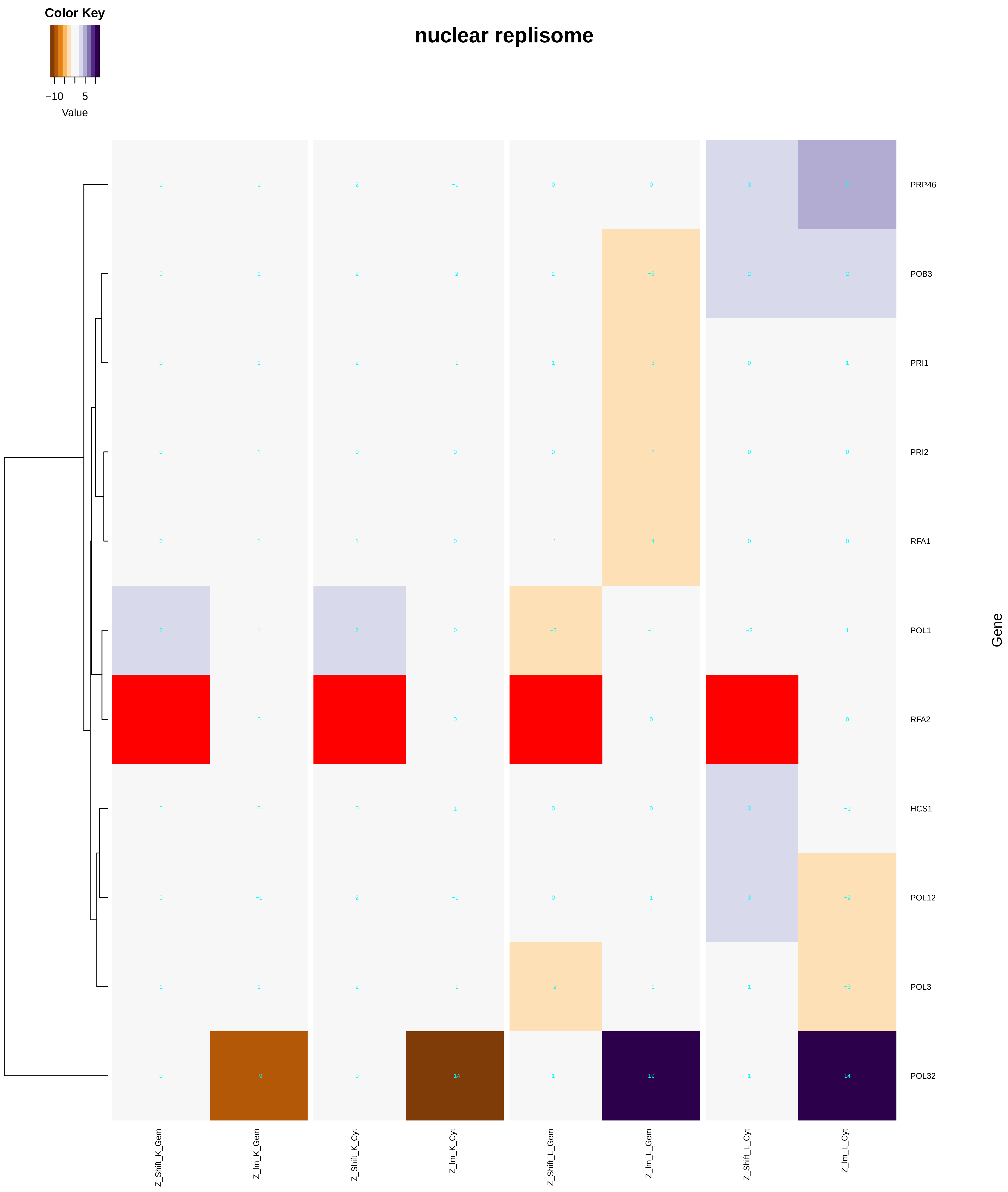
Color Key
−10
5
Value
nuclear replisome
PRP46
POB3
PRI1
PRI2
RFA1
POL1
RFA2
HCS1
POL12
POL3
POL32
Gene
Z_Shift_K_Gem
Z_Im_K_Gem
Z_Shift_K_Cyt
Z_Im_K_Cyt
Z_Shift_L_Gem
Z_Im_L_Gem
Z_Shift_L_Cyt
Z_Im_L_Cyt

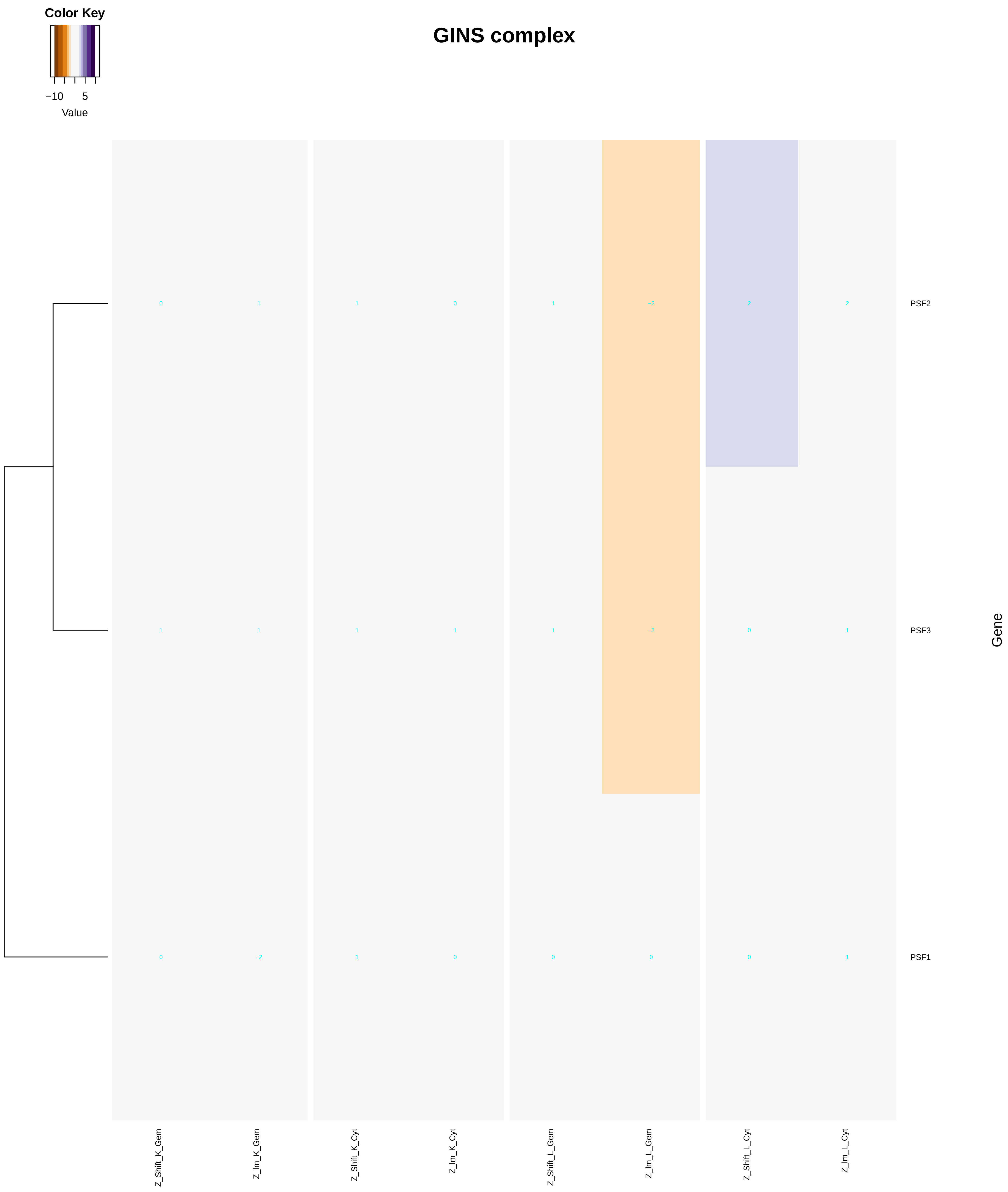

Color Key
−10
5
Value
GINS complex
PSF2
PSF3
PSF1
Gene
0 1 1 0 1 −2 2 2
1 1 1 1 1 −3 0 1
0 −2 1 0 0 0 0 1
Z_Shift_K_Gem
Z_Im_K_Gem
Z_Shift_K_Cyt
Z_Im_K_Cyt
Z_Shift_L_Gem
Z_Im_L_Gem
Z_Shift_L_Cyt
Z_Im_L_Cyt

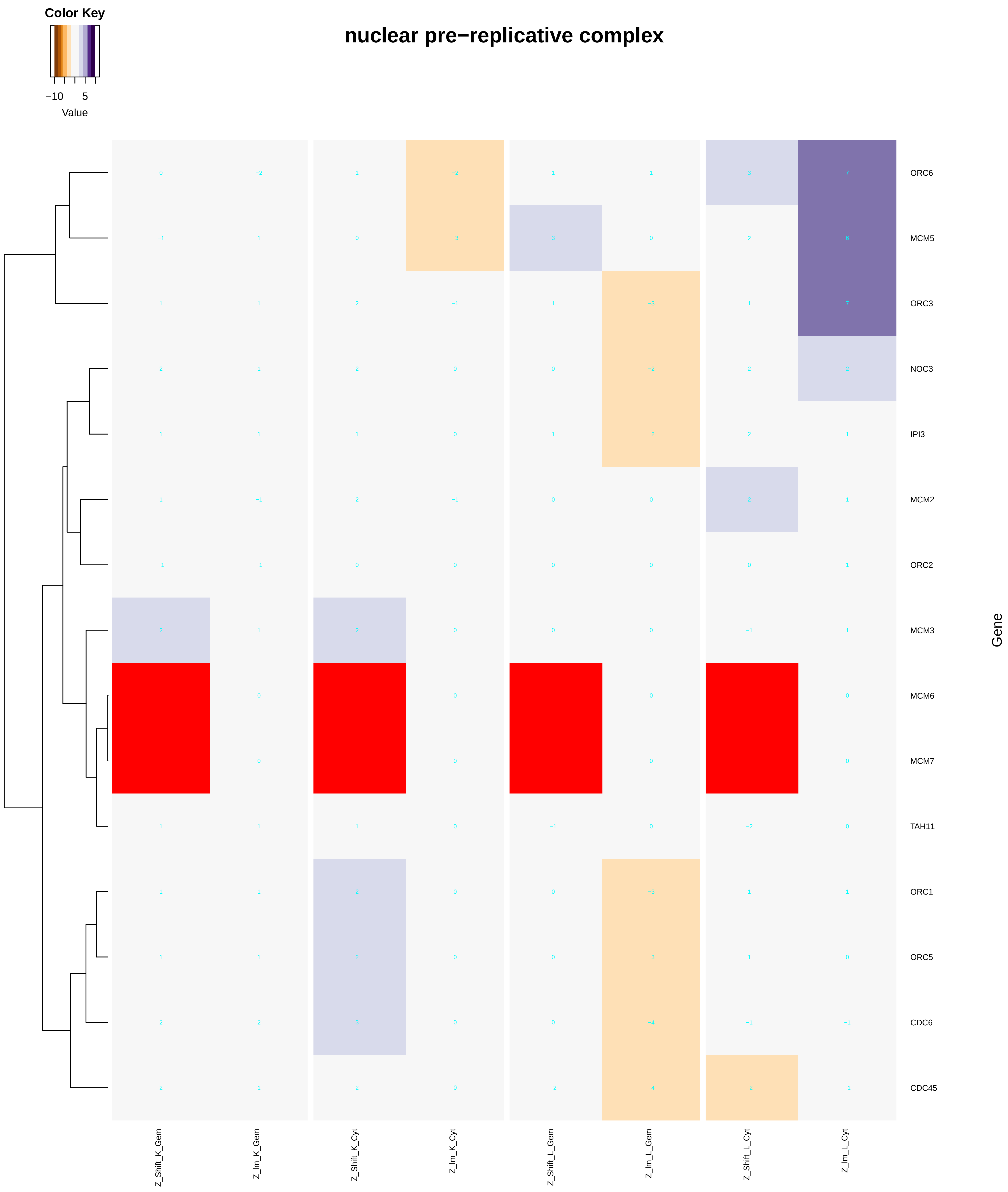
nuclear pre−replicative complex
Color Key
−10
5
Value
ORC6
MCM5
ORC3
NOC3
IPI3
MCM2
ORC2
MCM3
MCM6
MCM7
TAH11
ORC1
ORC5
CDC6
CDC45
Gene
Z_Shift_K_Gem
Z_Im_K_Gem
Z_Shift_K_Cyt
Z_Im_K_Cyt
Z_Shift_L_Gem
Z_Im_L_Gem
Z_Shift_L_Cyt
Z_Im_L_Cyt

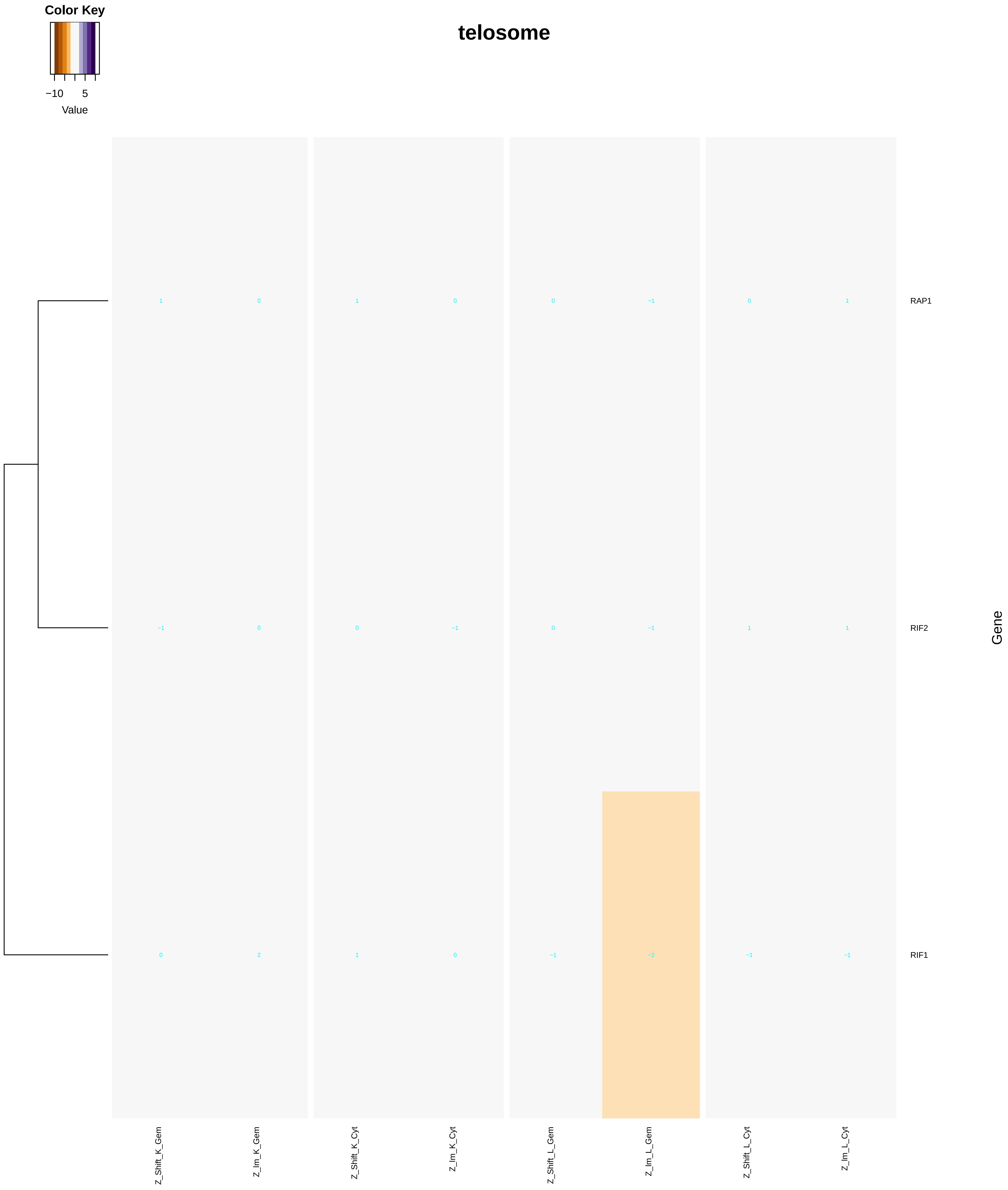

Color Key
−10
5
Value
telosome
1 0 1 0 0 −1 0 1
RAP1
−1 0 0 −1 0 −1 1 1
RIF2
0 2 1 0 −1 −2 −1 −1
RIF1
Gene
Z_Shift_K_Gem
Z_Im_K_Gem
Z_Shift_K_Cyt
Z_Im_K_Cyt
Z_Shift_L_Gem
Z_Im_L_Gem
Z_Shift_L_Cyt
Z_Im_L_Cyt

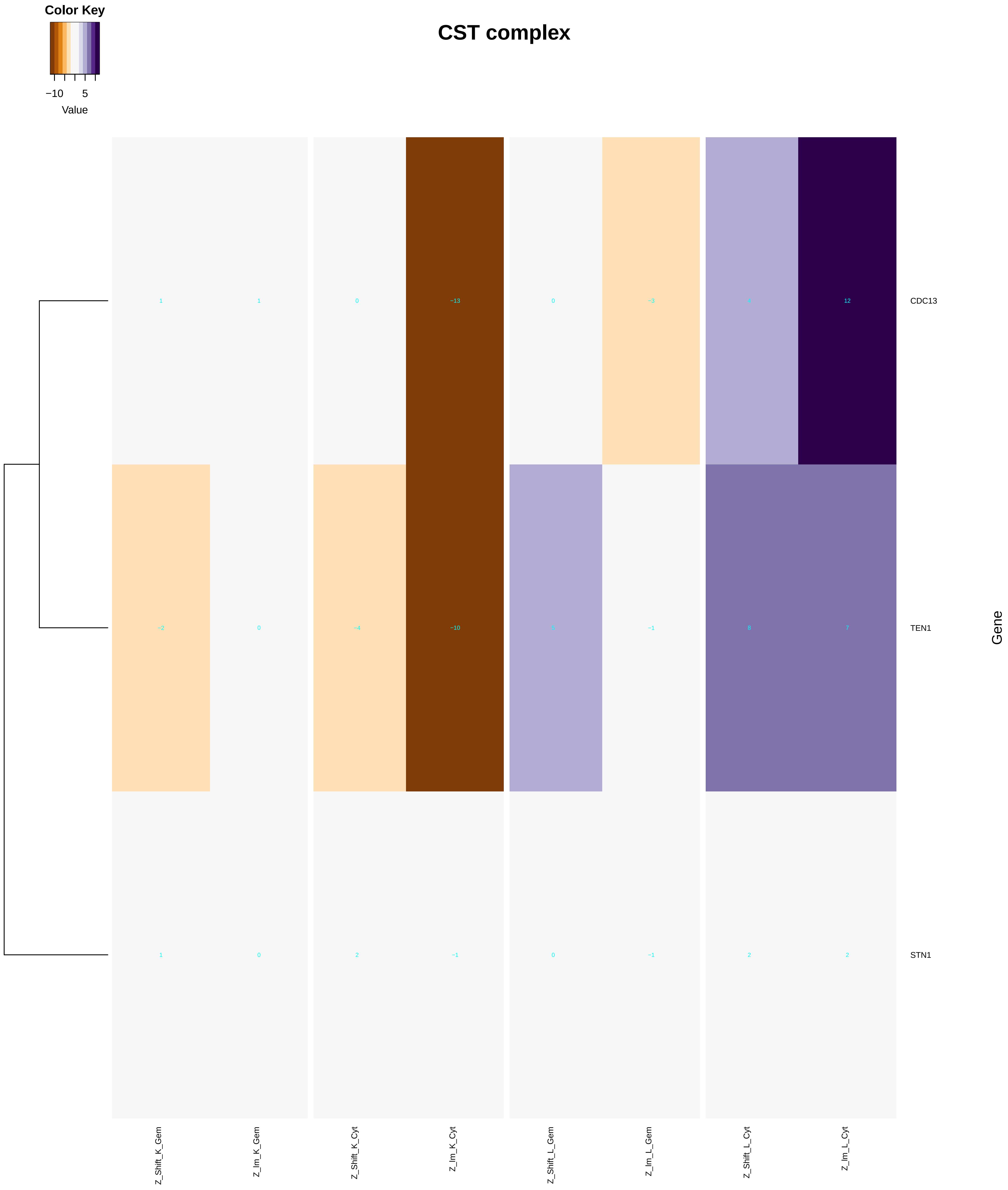

Color Key
−10
5
Value
CST complex
CDC13
TEN1
STN1
Gene
Z_Shift_K_Gem
Z_Im_K_Gem
Z_Shift_K_Cyt
Z_Im_K_Cyt
Z_Shift_L_Gem
Z_Im_L_Gem
Z_Shift_L_Cyt
Z_Im_L_Cyt
